# Optimizing Antibiotic Treatment for Diabetic Foot Infections: A Study From a Tertiary Public Healthcare Center in Puducherry, South India

**DOI:** 10.7759/cureus.60139

**Published:** 2024-05-12

**Authors:** Raghul Nagaya, Priyadharshini R, Reka Deva, Yogeshwari Jagadeesh, Pascal Emmanuel, Gaurang Narayan

**Affiliations:** 1 Internal Medicine, Jawaharlal Institute of Postgraduate Medical Education and Research, Puducherry, IND; 2 Pharmacology, Jawaharlal Institute of Postgraduate Medical Education and Research, Puducherry, IND; 3 General Surgery, Jawaharlal Institute of Postgraduate Medical Education and Research, Puducherry, IND; 4 Obstetrics and Gynecology, Indira Gandhi Government Medical College & Hospital, Nagpur, IND

**Keywords:** rational antibiotic use, microbial susceptibility, diabetic foot ulcer bacteria, diabetic foot infections, antibiotic prescribing pattern

## Abstract

Background: Diabetic foot infections (DFIs) represent a significant complication of diabetes mellitus, contributing to increased morbidity and mortality. Understanding antibiotic prescribing patterns and microbial susceptibility is crucial for effective management.

Objective: This study aimed to assess antibiotic prescribing trends and microbial susceptibility patterns in DFIs in a tertiary care center in Puducherry.

Methods: A prospective observational study was conducted over two months, involving patients with DFIs attending surgery OPD and admitted inpatient wards. Data on demographics, comorbidities, ulcer characteristics, antibiotic prescriptions, and microbial culture results were collected. Descriptive statistics and appropriate statistical tests were used for analysis.

Results: Of 110 patients included, most were males (80, 72.7%) aged 51-60 years (43, 39.1%). Common risk factors included poor glycemic control (85, 77.3%), barefoot walking (29, 26.4%), and a family history of diabetes (46, 41.8%). Gram-negative organisms (78, 70.9%) predominated, with *Escherichia coli *(17, 15.5%), *Pseudomonas aeruginosa* (12, 10.9%), and *Staphylococcus aureus* (10, 9.1%) being common isolates. Polypharmacy was observed, with (63) 57.3% receiving multiple antibiotics, mainly via the parenteral route (16, 64.5%). Ceftriaxone (31, 28.2%) and cefotaxime (21, 19.1%) were frequently prescribed. Antibiotic resistance varied among isolates.

Conclusion: This study underscores the predominance of gram-negative organisms in DFIs and highlights the need for rational antibiotic prescribing. Cephalosporins were commonly used, emphasizing the importance of empirical therapy. Understanding local microbial patterns and susceptibility is crucial for guiding antibiotic selection and optimizing clinical outcomes. In addition, addressing modifiable risk factors is imperative for preventing DFIs and reducing associated complications. This study provides valuable insights for strengthening antimicrobial stewardship programs and improving patient care in diabetic foot management. Furthermore, the present study highlights the importance of essentially deprescribing the prescriptions both from the patient, their primary carer, and the treating physician/surgeon's perspective.

## Introduction

Diabetes mellitus, a persistent metabolic condition, affects approximately 15% of the general population, with an estimated 19-34% of them experiencing diabetic foot ulcers during their lifetime [1-3]. Annually, between 9.1 and 26.1 million individuals worldwide are projected to develop diabetic foot ulcers, posing a significant challenge to healthcare systems due to prolonged hospital stays and poor healing outcomes despite standard care protocols [4-5].

While around 30% of patients witness wound healing within 12 weeks, a considerable 45% endure longer healing periods, with 25% of diabetes-related hospitalizations attributed to infected or ischemic diabetic foot ulcers [6-7]. These ulcers present severe complications, such as microthrombi formation, ischemia, necrosis, and gangrene, potentially leading to amputation. Effective antibiotic therapy is pivotal in managing diabetic foot infections (DFIs), where both monomicrobial and polymicrobial infections are prevalent [8].

A meta-analysis underscores the importance of infection prevention strategies and optimizing antibiotic therapy to curb overprescription, emphasizing the need for regular audits to monitor prescribing trends and bolster antibiotic stewardship efforts [9]. Recent studies highlight a concerning rise in drug-resistant strains, underscoring the necessity of tailoring antibiotic therapy based on resistance patterns to enhance healthcare outcomes [10].

Against this backdrop, our study aims to scrutinize antibiotic prescribing patterns and identify associated infection risk factors in a tertiary care setting. By shedding light on optimal antibiotic selection to mitigate resistance and complication risks, our findings will inform clinical decision-making and improve patient care outcomes.

## Materials and methods

The study was designed as a clinical, prospective observational investigation conducted over a two-month period at Jawaharlal Institute of Postgraduate Medical Education & Research (JIPMER), a central government public tertiary health care referral and teaching hospital in Puducherry, Southern India. Participants included patients with DFIs seen in the surgery OPD and those admitted to inpatient wards. Inclusion criteria specified patients aged over 30 years of either gender, diagnosed with DFIs, and prescribed at least one antibiotic, while exclusion criteria comprised pregnant or lactating women and those with foot infections due to chronic diseases, such as tuberculosis, malignancy, trauma, non-diabetic peripheral neuropathy, and arterial or venous disorders.

Sample size determination relied on previously published data, indicating an incidence of DFIs ranging between 8% and 10% in the Indian population [11]. The following formula was used:

$n = [Z^{2} \times p\times \ (1-p)] \div E^{2}$,

Where 𝑛n = sample size, 𝑍 = Z-score corresponding to the desired confidence level (e.g., for a 95% confidence level, 𝑍 = 1.96), 𝑝 = expected proportion or prevalence of the characteristic of interest (expressed as a decimal), and 𝐸 = absolute precision or margin of error (expressed as a decimal).

With an expected proportion of 8%, an absolute precision of 6%, and a 95% confidence level, the sample size was calculated to be 125 using convenience sampling.

The study was conducted between June 2022 and August 2022, after due ethical clearance (JIP/IEC-OS/2022/230). Written informed consent was obtained from all the participants in their local vernacular language Tamil. Demographic details and clinical findings were recorded, encompassing age, gender, education, occupation, socioeconomic status, physical activity or type of work, dietary habits, BMI, tobacco and alcohol use, family history of diabetes, HbA1c levels, concurrent conditions (e.g., diabetes mellitus, hypertension, hypothyroidism, and obesity), duration of diabetes mellitus and diabetic foot ulcer, antibiotics used, and antibiotic susceptibility patterns. All of these preliminary data were extracted from the existing medical records of the patients. As antibiotic susceptibility testing was concerned, for all the patients included in the study, Kirby-Bauer disk diffusion technique was the standard employed technique for determining the sensitivity patterns.

Data analysis involved input into Microsoft Excel version 20 (Microsoft Corporation, USA) and subsequent analysis using IBM SPSS Statistics for Windows, Version 22.0 (released 2013, IBM Corp., Armonk, NY). Descriptive statistics were used to describe demographic profiles, with continuous variables expressed as mean ± SD and categorical variables as frequency (n) and percentages (%). STROBE (Strengthening the Reporting of Observational Studies in Epidemiology) guidelines were adhered to in addition to duly incorporating the National and International Good Clinical Practices (GCP). Statistical tests were applied, considering a p-value <0.05 as significant.

## Results

A total of 110 patients diagnosed with diabetes mellitus and foot infections were recruited for this study. Table 1 shows the demographic characteristics of the study population. The majority 39%(43) of the participants were between 51 and 60 years of age, and 72.7% (80) of them were male. Few (n = 14, 12.7%) of the participants were graduates, while the majority (n = 22, 20%) had completed high school. Eighty-four (41.8%) of the participants were unemployed and belonged to either the lower middle class or lower class, according to modified Prasad's classification for socioeconomic status [12]. In addition, 26.4% (n = 29) had the habit of walking barefoot, and 41.8% (n = 46) had a family history of diabetes mellitus.

**Table 1:** Demographic characteristics of the study participants (n = 110)

Participant characteristics	Total patients (n = 110) n (%)
Gender	
Male	80 (72.7)
Female	30 (27.3)
Age (years)	
18-30	0 (0)
31-40	10 (9.1)
41-50	28 (25.5)
51-60	43 (39.1)
61-70	23 (20.9)
>70	6 (5.5)
Education	
Primary	20 (18.2)
Secondary	14 (12.7)
High school	22 (20)
Higher secondary	10 (9.1)
Graduation	14 (12.7)
Post graduation	0 (0)
Nil	30 (27.3)
Employment	
Working	64 (58.2)
Not working	84 (41.8)
Income (Modified Prasad's classification)	
Rs. 7770 and above (Upper class)	20 (18.2)
Between 3808 and 7769 (Upper middle class)	14 (12.7)
Between 2253 and 3808 (Middle class)	13 (11.8)
Between 1166 and 2253 (Lower middle class)	30 (27.3)
Below 1166 (Lower class)	33 (30)
Family H/o diabetes	
Yes	46 (41.8)
No	64 (58.2)
H/o Smoking and alcoholism	
Yes	8 (7.3)
No	102 (92.7)
H/o Barefoot walking	
Yes	29 (26.4)
No	81 (73.6)

Table 2 shows the diabetic-related characteristics in the study population. Almost half of the participants (n = 54, 49% of the patients) had diabetes mellitus for less than five years. Diabetes was poorly controlled (HbA1C > 8) in 77.3% (n = 85) of the cases. Most patients (n = 82, 74.5%) in the present study had foot ulcers for less than six months. Hypertension was the most commonly associated comorbid condition, seen in 24 patients. The majority of diabetic foot ulcers (n = 48, 43.6%) belonged to Wagner 2 category, followed by Wagner 1 (n = 30, 30%) and Wagner 3 (n = 29, 26.4%) [13]. Necrotizing soft tissue infection (NSTI) was present in more than half of the study population (57.3%, n = 63). While 19.1% (n = 21) of the participants had trophic ulcers, 8.2% (n = 9) presented with osteomyelitis. Nearly four-fifths of the patients (79.1%, n = 87) were on oral hypoglycemic agents, and 20.9% (n = 23) were on insulin. Nearly 1/5th of the subjects were on both OHA and insulin. Interventions like wound debridement were done in nearly 84.5% (n = 93) of the patients, and 15.5% (n = 17) underwent amputation.

**Table 2:** Diabetic foot-related demographic characteristics of the study participants (n = 110)

Participant characteristics	Total patients (n = 110) n (%)
Diabetes duration (yrs)	
< 5	54 (49.1)
6-10	32 (29.1)
11-15	14 (12.7)
16-20	5 (4.5)
21-25	3 (2.7)
>25	2 (1.8)
HbA1c levels	
Good control	17 (15.5)
Fair control	8 (7.3)
Poor control	85 (77.3)
Antidiabetic medications	
Oral hypoglycemic agents	87 (79.1)
Insulin	23 (20.9)
Diabetic foot ulcer duration	
<6 months	82 (74.5)
6 months-1 year	16 (14.5)
>1 year	12 (10.9)
Ulcer severity	
Mild	33 (30)
Moderate	48 (43.6)
Severe	29 (26.4)
Infection type	
NSTI (necrotizing soft tissue infection)	63 (57.3)
Cellulitis	11 (10.0)
NSTI (necrotizing soft tissue infection) with cellulitis	5 (4.5)
Trophic ulcer	21 (19.1)
Wet gangrene	1 (0.9)
Osteomyelitis	9 (8.2)
Treatment	
Amputation	17 (15.5)
Wound debridement	93 (84.5)
Concomitant diseases	
Hypertension	24 (24)
Epilepsy	2 (2.0)
Diabetic kidney disease	2 (2.0)
Hypothyroid	2 (2.0)
Nil	80 (80)

Table 3 illustrates the antibiotic prescription pattern. Gram-negative organisms were predominant in 70.9% (n = 78) of the specimens, followed by gram-positive organisms at 12.7% (n = 14). No growth was observed in 16.4% (n = 18) of the specimens. The prevalent isolates in this study were *Escherichia coli* (n = 17, 15.5%), *Pseudomonas aeruginosa* (n = 12, 10.9%), and *Staphylococcus aureus* (9.1%). Gram-negative organisms outnumbered gram-positive ones in terms of the number of isolated organisms. A majority (n = 63, 57.3%) of the participants received treatment with more than one antibiotic, and 64.5% (n = 71) were administered parenteral antibiotics. The most frequently prescribed antibiotics included ceftriaxone (n = 31, 28.2%) and cefotaxime (n = 21, 19.1%), with common combinations being piperacillin with tazobactam (n = 11, 10%) and amoxicillin with clavulanic acid (n = 21, 19.1%). Other antibiotics prescribed included cefoperazone sodium with sulbactam, as well as ciprofloxacin, clindamycin, linezolid, amoxicillin, gentamycin, and amikacin.

**Table 3:** Antibiotic prescribing pattern among the study participants (n = 110)

Participant characteristics	Total patients (n = 110) n (%)
Antibiotics prescribed	
Cefotaxime	21 (19.1)
Ceftriaxone	31 (28.2)
Cefoperazone and sulbactam	4 (3.6)
Clindamycin	1 (0.9)
Amikacin	1 (0.9)
Amoxicillin and clavulanic acid	21 (19.1)
Piperacillin and tazobactam	11 (10)
Ciprofloxacin	5 (4.5)
Metronidazole	2 (1.8)
Linezolid	5 (4.5)
Cloxacillin	8 (7.3)
No. of antibiotics prescribed	
Single	47 (42.7)
Multiple	63 (57.3)
Antibiotic formulations prescribed	
Oral	34 (30.9)
Parenteral	71 (64.5)
Both	5 (4.5)
Microbiological culture report	
No bacteria	18 (16.4)
Gram positive	14 (12.7)
Gram negative	78 (70.9)
Distribution of gram-positive and gram-negative organisms	
Acinetobacter baumannii	16 (14.5)
*Aeromonas* sp.	1 (0.9)
*Citrobacter* sp.	1 (0.9)
Enterobacter cloacae	4(3.6)
Enterococcus faecalis	3 (2.7)
Entercoccus faecium	1 (0.9)
Escherichia coli	17 (15.5)
Klebsiella pneumoniae	10 (9.1)
Proteus mirabilis	11 (10)
Providencia stuartii	2 (1.8)
Pseudomonas aeruginosa	12 (10.9)
Staphylococcus aureus	10 (9.1)
Streptococcus agalactiae	4 (3.6)
No organism	18 (16.4)

Table 4 shows the antibiotic sensitivity and resistance pattern. Different organisms show different sensitivity and resistance patterns.

**Table 4:** Antibiotic susceptibility pattern among organisms isolated.

Organisms Isolated (n)	Most susceptible antibiotic	Most resistant antibiotic
*Escherichia coli* (17)	Tigecycline	Ceftazidime co-trimoxazole
*Proteus vulgaris* (11)	Amikacin piperacillin and tazobactam	Ciprofloxacin
*Klebsiella pneumoniae *(10)	Tigecycline piperacillin and tazobactam	Ceftriaxone, ceftazidime, ciprofloxacin
*Pseudomonas aeruginosa *(12)	Amikacin meropenem	Ceftriaxone and ceftazidime
*Methicillin-resistant Staphylococcus aureus* (*MRSA*) (2)	Linezolid	Gentamycin, norfloxacin, and erythromycin
*Streptococcus* sp.	Meropenem linezolid	Erythromycin and ceftriaxone

## Discussion

The current study aimed to assess the antibiotic prescribing pattern in DFI patients and evaluate the antimicrobial susceptibility of the involved microbes. Over a two-month period, 110 patients diagnosed with DFIs were enrolled in this descriptive observational study. The demographic profile revealed that the majority (n = 43, 39%) of the participants fell within the 51-60 age range, with males constituting 72.7% (n = 80) of the sample, aligning closely with previous research [14]. Male predominance in DFIs is often linked to their occupational activities requiring outdoor exposure [11,15-16]. Risk factors identified in our study included low socioeconomic status, barefoot walking habits, and poor glycemic control (HbA1C > 8%), consistent with findings from other populations [7,11,16].

Notably, 49% (n = 54) of the patients had diabetes for less than five years, contrasting with the findings of Gadepally et al., emphasizing the importance of strict glycemic control for infection prevention, which may necessitate long-term management [17]. Most patients (74.5%, n = 82) had foot ulcers for less than six months, consistent with previous reports [18]. Hypertension emerged as the most prevalent comorbidity, in line with the findings of Jothylekshmy et al. [19].

The severity of foot ulcers varied, with Wagner 2 lesions comprising the majority (43.6%, n = 48), followed by Wagner 1 (30%, n = 33) and Wagner 3 (26.4%, n = 29). Necrotizing soft tissue infection (NSTI) was observed in 57.3% (n = 63) of cases, while trophic ulcers and osteomyelitis affected 19.1% (n = 21) and 8.2% (n = 9) of the participants, respectively. Treatment modalities included oral hypoglycemic agents (79.1%, n = 87) and insulin (20.9%, n = 23), with 84.5% (n = 93) of patients undergoing wound debridement and 15.5% (n = 17) requiring amputation, mirroring previous findings [11,17,19-20].

Microbiological analysis revealed gram-negative organisms predominating (70.9%, n = 78), notably *E. coli* (15.5%, n = 17) and *Pseudomonas aeruginosa* (10.9%, n = 12), followed by *Staphylococcus aureus* (9.1%, n = 10). Polypharmacy was common, with 57.3% (n = 63) of the participants receiving multiple antibiotics, and 64.5% (n = 71) administered parenteral antibiotics. Predominant antibiotic choices included ceftriaxone (28.2%, n = 31) and cefotaxime (19.1%, n = 21), with combinations like piperacillin with tazobactam (10%, n = 11) and amoxicillin with clavulanic acid (19.1%, n = 21) also prevalent. Other antibiotics such as ciprofloxacin, clindamycin, linezolid, amoxicillin, gentamicin, and amikacin were used. These findings are also consistent with existing literature [8,11,21-22]. Furthermore, the point on polypharmacy correctly highlights the importance of essentially deprescribing or prescription pruning seconding the findings of Narayan et al. [23].

Integration of findings from additional studies provides further insights into the complexity of diabetic foot complications. Research conducted among Saudi Arabians highlighted the commonality of foot ulcers, amputations, gangrene, and bone deformities among older males with poorly controlled diabetes. This underscores the importance of addressing not only the infection but also the broader spectrum of complications associated with diabetic foot [24].

Moreover, advancements in artificial intelligence (AI) present opportunities to enhance pharmacological research, including drug discovery, development, and personalized medicine. AI algorithms offer valuable tools for analyzing biomedical data, predicting drug efficacy, and optimizing treatment strategies. However, ethical considerations such as data privacy, algorithm bias, and human oversight are paramount in the responsible deployment of AI in healthcare [25]. Furthermore, prospective studies like the one conducted in Pakistan emphasize the therapeutic challenge of treating diabetic foot ulcers, particularly in regions with limited healthcare resources. Early surgical intervention and appropriate medical care are crucial in improving outcomes and reducing the burden of diabetic foot complications [26].

The study's strengths lie in its comprehensive approach, robust sample size, diverse participant profile, alignment with existing literature, detailed microbiological analysis, and insights into treatment modalities, all of which contribute to a thorough understanding of antibiotic prescribing patterns and antimicrobial susceptibility in DFIs. Although our study offers valuable insights, limitations include its short duration and the lack of long-term follow-up to assess outcome variations and potential changes in antibiotic prescribing trends.

In summary, a multidimensional approach, integrating clinical, microbiological, and technological advancements, is essential in addressing the complexities of DFIs. This includes not only optimizing antibiotic prescribing patterns but also addressing broader healthcare challenges through interdisciplinary collaboration and ethical deployment of innovative technologies like AI.

## Conclusions

This study aimed to provide preliminary insights into the prescription patterns of antibiotics and the susceptibility of microbes in DFIs, aiding in the selection of appropriate antibiotic therapy. Our findings revealed a predominance of gram-negative organisms, particularly *E. coli*, isolated from DFIs. Cephalosporins, such as ceftriaxone and cefotaxime, were commonly prescribed for treatment, with a majority of patients undergoing parenteral antibiotic therapy. Risk factors associated with DFIs included low socioeconomic status, barefoot walking, poor glycemic control, and a strong family history of diabetes mellitus. It is noteworthy that different organisms exhibit varying patterns of sensitivity and resistance, underscoring the importance of tailoring antibiotic selection to the microbial patterns specific to each region and the antimicrobial susceptibility observed. Such tailored approaches hold promise in reducing the morbidity and mortality associated with DFIs and contribute to a deeper understanding of antibiotic resistance dynamics, thus reinforcing the imperative for judicious antibiotic therapy selection and bolstering antimicrobial stewardship efforts. Furthermore, the present study highlights the importance of essentially deprescribing the prescriptions both from the patient, their primary carer, and the treating physician/surgeon's perspective.
